# Long noncoding RNA KCNMA1-AS1 promotes osteogenic differentiation of human bone marrow mesenchymal stem cells by activating the SMAD9 signaling pathway

**DOI:** 10.1186/s13062-023-00425-2

**Published:** 2023-11-29

**Authors:** Zhaoyi Mai, Jingpeng Liu, Xiao Jiang, Wenli Gu, Wei Wang, Simin Li, Gerhard Schmalz, Hui Xiao, Jianjiang Zhao

**Affiliations:** 1 Stomatological Hospital, School of Stomatology, Southern Medical University, Guangzhou, Guangdong China; 2 Department of Cariology, Endodontology and Periodontology, University of Leipzig, 04103 Leipzig, Germany; 3 Shenzhen Stomatological Hospital, Southern Medical University, Shenzhen, Guangdong China

**Keywords:** Human bone marrow mesenchymal stem cells, Osteogenic differentiation, lncRNA, KCNMA1-AS1, SMAD9

## Abstract

The human bone marrow mesenchymal stem cells (hBMSCs) undergo intense osteogenic differentiation, a crucial bone formation mechanism. Evidence from prior studies suggested an association between long noncoding RNAs (lncRNAs) and the osteogenic differentiation of hBMSCs. However, precise roles and molecular mechanisms are still largely unknown. In this work, we report for the first time that lncRNA KCNMA1 antisense RNA 1 (KCNMA1-AS1) plays a vital role in regulating hBMSCs’ osteogenic differentiation. Here, it was observed that the KCNMA1-AS1 expression levels were significantly upregulated during osteogenic differentiation. In addition, KCNMA1-AS1 overexpression enhanced in vitro osteogenic differentiation of hBMSCs and in vivo bone formation, whereas knockdown of KCNMA1-AS1 resulted in the opposite result. Additionally, the interaction between KCNMA1-AS1 and mothers against decapentaplegic homolog 9 (SMAD9) was confirmed by an RNA pull-down experiment, mass spectrometry, and RIP assay. This interaction regulated the activation of the SMAD9 signaling pathway. Moreover, rescue assays demonstrated that the inhibitor of the SMAD9 signaling pathway reversed the stimulative effects on osteogenic differentiation of hBMSCs by KCNMA1-AS1 overexpression. Altogether, our results stipulate that KCNMA1-AS1 promotes osteogenic differentiation of hBMSCs via activating the SMAD9 signaling pathway and can serve as a biomarker and therapeutic target in treating bone defects.

**Supplementary Information:**

The online version contains supplementary material available at 10.1186/s13062-023-00425-2.

## Introduction

The bone tissue structure of the oral and maxillofacial region is an important basis for supporting the facial shape. However, maxillofacial bone defects due to trauma, tumor resection, infection, and congenital malformation can cause not only facial deformities, but also difficulty in chewing, swallowing, speaking, and breathing. This could affect a patient’s physiological and psychological well-being [1–3]. Nowadays, autologous bone grafts are commonly employed as the standard procedure to repair bone defects. Yet, the disadvantages such as secondary injury of the donor site, limited supply, and poor prognosis in patients with osteoporosis are prominent [4, 5]. Although synthetic bone can be an alternative, its limitations include immune rejection, infection, and low capacity for osteoinduction and osteo-integration [6, 7]. Lately, bone tissue engineering has been the focus and is receiving extensive attention and research. Bone tissue engineering is a combination of seed cells, specific osteogenic factors, and biological scaffold materials aiming to create a reconstructed tissue with a similar function as a natural bone tissue; it is anticipated to offer a successful procedure for bone reconstruction [8–10]. Among the various types of cells, mesenchymal stem cells (MSCs) are generally preferred for bone repair. Human bone marrow mesenchymal stem cells (hBMSCs), as adult stem cells, have promising potential as seed cells in bone tissue engineering owing to their ability to self-renew, pluripotency, and mild immunogenicity [11–13]. Osteogenic differentiation of MSCs primarily includes cell differentiation, cell maturation, extracellular matrix formation, and mineralization. This process can be regulated by growth factors belonging to the Wnt family and bone morphogenetic protein (BMP) family, transcription factors that include β-catenin, runt-related transcription factor 2 (RUNX2), osterix (OSX), and certain microRNAs (miRNAs) [14, 15].

In some recent studies, long noncoding RNAs (lncRNAs) are reported to regulate how hBMSCs differentiate into osteoblasts [16, 17]. LncRNAs are noncoding RNAs (ncRNAs) typically characterized by > 200 nucleotides in length; they were previously described as “transcriptional noise” and regarded as ineffectual RNAs [18–20]. Nevertheless, several lncRNAs contain conserved secondary and higher-order structures, suggesting their prospective roles in gene regulation [21]. The total number of known lncRNA continues to increase as the next-generation RNA sequencing technologies are increasingly being used.

lncRNAs have emerged as important regulators in various diseases, including inflammatory diseases and cancers. In the context of the inflammatory diseases, studies by Ashrafizadeh et al. [22] and Chew et al. [23] demonstrated that lncRNAs can positively or negatively regulate inflammatory pathways (NFκB pathway, p38 MAPK pathway, and JAK/STAT pathway) by influencing the expression of critical genes involved in inflammation (e.g., Pro-inflammatory cytokines (interleukin-6 (IL-6), tumor necrosis factor-alpha (TNF-α), and interleukin-1β (IL-1β)), Chemokines (C-C motif chemokine ligand 2 (CCL2) and C-X-C motif chemokine ligand 8 (CXCL8)), Adhesion molecules (intercellular adhesion molecule 1 (ICAM-1) and vascular cell adhesion molecule 1 (VCAM-1)), transcription factors (NFκB, AP-1, and STATs)). As an example, Akıncılar et al. described a specific lncRNA called NAIL that is essential for the coordinated activation of p38 and NFκB pathways in colitis, highlighting the importance of lncRNAs in inflammatory bowel diseases (IBDs) [24]. In the context of cancer, lncRNAs have also been implicated in regulating cancer stem cells (CSCs). Ma et al. [25] discussed the expanding roles of lncRNAs in the regulation of CSCs, which are a small population of cells within tumors that possess self-renewal and tumor-initiating properties. LncRNAs have been shown to regulate various aspects of CSC biology, including self-renewal, metastasis, and drug resistance. LncRNAs have the potential to serve as diagnostic biomarkers and therapeutic targets, offering new opportunities for the development of novel treatment strategies in various diseases including inflammatory diseases and cancers.

Additionally, many studies demonstrate that dysregulated lncRNAs are implicated in major diseases including osteoporosis and skeletal aging [26–30]. Functionally, lncRNAs can act as competing endogenous RNA (ceRNA) with miRNA forming a regulatory network through the lncRNA/miRNA/mRNA signaling axis [31, 32]. Meanwhile, lncRNA can also be involved in the nuclear scaffold formation, regulation of protein and RNA stability, and transcriptional modulation [33, 34]. Studies have identified massive lncRNAs that are differentially expressed during osteogenesis, which include H19 [35, 36], MALAT1 [36], DANCR [37, 38], FAM83H-AS1 [39](Wu, Cao et al. 2020), and MCF2L-AS1 [39]. These have been further confirmed to regulate osteogenic markers, key regulators and signaling pathways in osteogenic differentiation. Even though the reports about lncRNAs keep emerging, how lncRNAs delicately orchestrate osteogenic differentiation of hBMSCs remains greatly enigmatic and needs to be probed deeper.

Located in the chromosome region 10q22.3, lncRNA KCNMA1 antisense 1 (KCNMA1-AS1) was initially demonstrated to promote the progression and migration of epithelial ovarian cancer (EOC) via the apoptosis pathway [25]. However, the association between KCNMA1-AS1 and osteogenic differentiation of hBMSCs has not yet been explained. Thus, in this study, we first revealed that KCNMA1-AS1, whose upregulated expression was observed during osteogenesis, is an essential component, whereas hBMSCs undergo osteogenic differentiation. Further explorations elucidated that KCNMA1-AS1 interacts with mothers against decapentaplegic homolog 9 (SMAD9) and facilitates hBMSCs’ osteogenic differentiation by activating the SMAD9 signaling pathway. These findings indicate the promising potential of KCNMA1-AS1 as a newly discovered therapeutic target for the restoration of bone.

## Materials and methods

### Cell culture

hBMSCs were supplied by Cyagen Biosciences (Guangzhou, China). The attached flow cytometry report shows that hBMSCs positively expressed CD29, CD44, CD73, and CD105, and negatively expressed CD11b, CD34, and CD45. Under specific inductive conditions, hBMSCs differentiate into adipocytes, chondrocytes, and osteocytes. hBMSCs were cultured in hBMSCs basal medium by Cyagen Biosciences, Guangzhou, China following their protocols (supplemented with fetal bovine serum or FBS- 10%, glutamine- 1%, and penicillin-streptavidin solution- 1%). hBMSCs were seeded in 6-well plates for osteogenic differentiation (density ~ 1 × 10^5^ per well). Upon reaching a cell confluency of 70%, an osteogenic differentiation medium (Cyagen Biosciences, Guangzhou, China) was used instead of the culture medium. The osteogenic differentiation medium consists of 10% FBS, 10mM β-glycerophosphate, 0.05mM ascorbic acid, and 100nM dexamethasone. The cultures were placed in a humid incubator with 5% CO2 and temperature maintained at 37 °C. The medium was replaced every 3 days.

### RNA extraction and quantitative real-time PCR

RNAex Pro Reagent by Accurate Biology, Hunan, China was used to extract the whole RNA as instructed by the manufacturers. The concentration and purity of each RNA sample were evaluated by the A260/A280 ratio on a NanoDrop 2000 instrument (ThermoFisher, USA). With the help of Evo M-MLV RT Kit with gDNA Clean for qPCR (Accurate Biology, Hunan, China), reverse transcription was carried out. Quantitative real-time PCR (qPCR) was done using a CFX Connect Real-Time PCR Detection System (BioRad, USA) with SYBR Green Premix Pro Taq HS qPCR Kit (Accurate Biology, Hunan, China) following the product instructions. Reactions were programmed at 95℃ for 30 s, followed by 40 cycles of 95℃ and 63℃ for 5 and 30 s, respectively. Each sample was analyzed in triplicates. GAPDH expression was used as a reference for KCNMA1-AS1 and other genes. The relative expression level of each gene was calculated by normalizing it to GAPDH expression levels, for which the 2^−ΔΔCt^ method was used. Tsingke Biotechnology (Beijing, China) helped design and synthesize the primers we used in the present research (Supplementary File: Table S1).

### Western blotting

The RIRA lysis buffer (Cowin Biotech, Jiangsu, China) together with a phosphatase inhibitor cocktail and a protease inhibitor cocktail (Cowin Biotech, Jiangsu, China), was used to extract whole protein from the cells. The concentration of each protein sample was measured using a BCA protein assay kit (Cowin Biotech, Jiangsu, China) as per the instructions given by the manufacturer. SDS-PAGE with a total of 15 µg protein per lane was performed followed by transferring the resulting lanes onto a polyvinylidene difluoride (PVDF) membrane (Millipore, USA). After blocking with 5% bovine serum albumin (BSA) in TBST (0.1% Tween-20 added to TBS) for 1.5 h at room temperature, the membranes were treated overnight at 4 °C with the primary antibodies. They were then washed with TBST three times before being incubated with the secondary antibodies (Goat Anti-Rabbit IgG or Goat Anti-Mouse IgG, 1:5000, Proteintech, Wuhan, China) that were conjugated with HRP (horseradish peroxidase) for 1 h at room temperature. The membranes were visualized in a chemiluminescence imaging system (Uvitec, UK) using a chemiluminescent HRP substrate (Millipore, USA). The density of the bands was analyzed using the ImageJ software (National Institute of Health, NIH, USA). Primary antibodies are listed as follows: COL1A1 (1:1000, Abmart, Shanghai, China), RUNX2 (1:1000, Cell Signaling Technology, USA), Osterix (1:1000, Boster, Wuhan, China), OPN (1:1000, Abcam, UK), SMAD9 (1:500, Abmart, Shanghai, China), P-SMAD9 (1:1000, Affinity, Jiangsu, China), GAPDH (1:10000, Proteintech, Wuhan, China).

### Staining with Alkaline phosphatase (ALP) and ALP activity assay

The seeded hBMSCs were subjected to culturing in an osteogenic differentiation medium. After seven days, the cells were harvested, washed thrice with phosphate buffer saline (PBS), fixed in 4% paraformaldehyde for half an hour, and washed three times with ultrapure water. Staining was done using the ALP staining kit by Beyotime Biotechnology, Shanghai, China as per their provided protocols. Images were obtained from an inverted microscope system (Leica, Germany). For ALP activity assay, following guidelines, hBMSCs were covered with lysis buffer without inhibitors (Beyotime Biotechnology, Shanghai, China) and measured using an ALP assay kit (Beyotime Biotechnology, Shanghai, China) following guidelines. Absorbance at 405 nm was calculated for each sample in a microplate reader (BioTek, USA). Each sample was analyzed in triplicate.

### Alizarin Red S (ARS) staining and ARS quantification assay

The seeded hBMSCs in 6-well plates were cultured in an osteogenic differentiation medium for two weeks. After three PBS washes, the harvested cells were fixed in 4% paraformaldehyde, washed thrice with ultrapure water, and stained with ARS solution (ScienCell, USA) as per instruction given by the manufacturers. An inverted microscope system (Leica, Germany) was used to capture the images. The stained cells were then collected and quantified using the ARS assay kit by ScienCell, USA, following the product’s instructions. The absorbance_405nm_ was determined on a microplate reader (BioTek, USA). Each sample was analyzed in triplicate.

### Lentivirus construction and cell transfection

KCNMA1-AS1 sequences were amplified and inserted into the GV502 vector for KCNMA1-AS1 overexpression (named KCNMA1-AS1), and a blank vector served as the negative control (NC). siRNA (Small interfering RNA) targeting KCNMA1-AS1 and its scrambled control were synthesized and converted into GV248 vector, named short hairpin (sh) -KCNMA1-AS1 and sh-NC respectively. The procedures above and lentivirus construction were accomplished by GeneChem (Shanghai, China). Lentivirus was then transfected into hBMSCs at MOI of 40 for 72 h. Stable transfection was screened by culturing hBMSCs in a normal medium with 2 µg/mL puromycin (Solarbio, Beijing, China). The siRNA sequences used are listed in **Supplementary File: Table S2**.

### Animal experiments

About 4-week-old immunodeficient female BALB/c nude mice were obtained commercially from Guangdong Medical Laboratory Animal Center (GDMLAC, Foshan, China). They were randomly classified into four groups of 5 mice each. To establish the xenograft models, hBMSCs were grown for 7 days in an osteogenic differentiation medium. Approximately 1 × 10^7^ cells were collected and loaded onto 40 mg hydroxyapatite (HAP, BioDuly, Nanjing, China) and subcutaneously implanted onto the dorsal part of BALB/c nude mice under general anesthesia with isoflurane. After four weeks, euthanasia was executed by cervical dislocation under general anesthesia with excessive isoflurane. Xenografts were surgically removed following euthanasia. They were fixed using 4% paraformaldehyde for 48 h and were decalcified for 28 days using EDTA (pH = 7.2). The Animal Ethical and Welfare Committee of GDMLAC (C202207-9) reviewed and approved all animal experiments.

#### Micro CT analysis

The xenografts fixed in 4% paraformaldehyde were scanned in a Micro CT system (Bruker, Germany). The system was set at Al 0.5 mm filter mode and the highest resolution in this mode was adjusted to 8 μm. The X-ray source worked at 70 kV, 200 µA, and the exposure time was 460 ms. The matching NRecon software was used to reconstruct the two-dimensional sections of the original scanned Micro CT data, and the reconstruction scope was selected to include the whole sample with appropriate correction. The data of the two-dimensional sections were quantitatively analyzed using the supporting CTan analysis software, and the analysis area of each layer (ROI, region of interest) was drawn according to the tissue images of subcutaneous implants; subsequently, the VOI (volume of interest) was formed by multi-layer stacking. The applicable threshold was modified to dualize the calcified tissue in the selected area. The mineral density of the bone was analyzed and the bone morphometry of calcified tissues was processed after binary treatment.

### Morphological staining and immunohistochemistry

Decalcified xenografts of nude mice were kept in turn through gradient ethanol for dehydration and xylene for transparency. The xenografts were then paraffin-embedded and sectioned into slices of 4 μm thickness each. Subsequently, the sections were treated with xylene to de-paraffinize; and rehydrated by graded alcohol treatment. Hematoxylin-eosin (HE) staining and Masson’s trichrome staining were essential for morphological observations; they were performed using the HE staining kit and Masson’s trichrome staining kit (Servicebio, Wuhan, China), respectively, following the instruction provided by the manufacturer. For immunohistochemistry (IHC), the sections were treated with citrate buffer for antigen retrieval, followed by 30 min of blocking with 3% BSA at room temperature and the final overnight incubation at 4 °C with the primary antibodies. The slices were subjected to treatment with secondary antibodies conjugated with HRP (Goat Anti-Rabbit IgG or Goat Anti-Mouse IgG, 1:500, Biosharp, Anhui, China) following three PBS washes for 1 h each at room temperature. Per the product’s instructions, they were visualized using a 3,3’-diaminobenzidine (DAB) chromogenic kit (Servicebio, Wuhan, China). An upright microscope system (Mshot, Guangzhou, China) captured the images. Primary antibodies are listed as follows: COL1A1 (1:100, Boster, Wuhan, China), RUNX2 (1:100, Santa Cruz, USA), OPN (1:100, Abcam, UK), SMAD9 (1:100, Bioss, Beijing, China), phosphorylated SMAD9 (1:100, Affinity, Jiangsu, China).

### Fluorescence in situ hybridization (FISH) and immunofluorescence

Axl-bio, Guangzhou, China helped design and synthesize probes that were labeled with FAM or TAMRA targeting KCNMA1-AS1 and the probe of its negative control labeled with FAM. Six-well plates with cell slides were used for seeding the hBMSCs maintaining a density of 6 × 10^4^ per well; they were then fixed for 30 min in 4% paraformaldehyde. Fluorescence in situ hybridization (FISH) was performed using the FISH assay kit (Axl-bio, Guangzhou, China). The primary antibodies (SMAD9, 1:200, Bioss, Beijing, China) were incubated on slides used for immunofluorescence (IF) at 4 °C overnight after its blocking with 1% BSA. The slides were incubated for an hour at room temperature with the fluorescein isothiocyanate (FITC)-conjugated goat anti-rabbit secondary antibodies (1:200, Proteintech, Wuhan, China) after three PBST (0.1% Tween-20 added to PBS) washes. With the aid of a confocal laser scanning microscope (Carl Zeiss, Germany), fluorescence was stimulated and recorded. The probe sequences are listed in **Supplementary File: Table S3**.

### RNA pull-down assay and mass spectrometry

A biotin-labeled probe of KCNMA1-AS1 was purchased from Axl-bio (Guangzhou, China); a probe against LacZ mRNA (LacZ) was used as a negative control. Biotinylated and negative control probes (2–5 µg) were collected and mixed with RNA structure buffer to form an appropriate RNA secondary structure. The pre-treated mixtures were incubated with streptavidin magnetic beads at 25℃ for 30 min, then gently incubated with lysates of hBMSCs on a rotator for 2 h at 25℃. Gel electrophoresis was conducted to separate the pull-down products released from the beads following elution; the gels were visualized by silver staining and used for mass spectrometry (Q Exactive, Thermo Fisher, USA). Proteins were identified using the Mascot Server (Matrix Science, UK). The probe sequences are listed in Supplementary File: Table S4. The images from silver staining have been presented in Supplementary File: Figure S1.

### RNA immunoprecipitation assay

Using a RIP kit (Axl-bio, Guangzhou, China) and its product manual, an RNA immunoprecipitation (RIP) assay was performed. In short, the collected hBMSCs were resuspended in polysome lysis buffer containing a protease inhibitor and an RNase inhibitor. The supernatants collected after centrifugation were incubated with indicated antibodies (SMAD9, LifeSpan Biosciences, USA) and control protein rabbit IgG (Cell Signaling Technology, USA) with gentle agitation at 4℃ overnight; it was lightly incubated with prepared protein A/G beads on a vertical mixer at 4℃ for 1 h. RNA extracted from the beads was measured by qPCR. The electrophoresis gel of RIP assay has been presented in Supplementary File: Figure S2.

### Statistical analysis

Data from at least three distinct experiments was collected and presented as mean ± standard deviation (SD). Pearson’s Correlation Coefficient was used to evaluate the association between any two variables. However, an unpaired Student’s *t*-test was used to compare the variations between the groups. A *P-*value of *<* 0.05 indicated the significance level. This study performed all the statistical analyses using the GraphPad Prism 8 (GraphPad Software, USA) software.

## Results

### Upregulation of KCNMA1-AS1 is observed during the osteogenic differentiation of hBMSCs

**Fig. 1:**
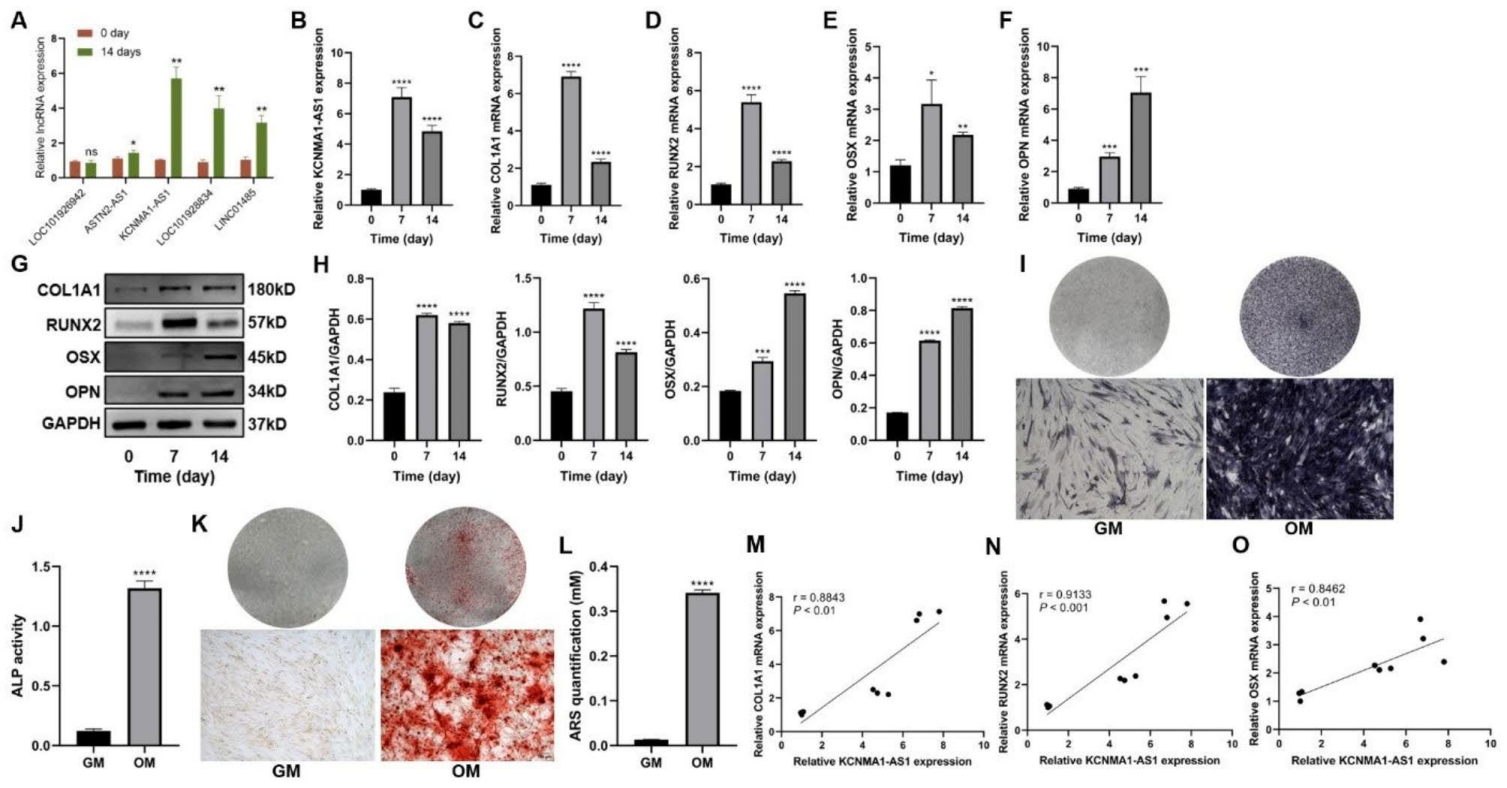
KCNMA1-AS1 is upregulated when hBMSCs undergo osteogenic differentiation. **A-** Differentially expressed lncRNAs analyzed by qPCR after 14 days of osteogenic induction. **B-** Relative expression levels of KCNMA1-AS1 were measured using qPCR, and Glyceraldehyde 3-phosphate dehydrogenase (GAPDH) was used for normalization. **C-F-** Relative mRNA levels of COL1A1 (**C**), RUNX2 (**D**), OSX (**E**), and OPN (**F**) measured through qPCR, normalized to GAPDH. **G** and **H-** The protein levels of COL1A1, RUNX2, OSX, and OPN were detected by western blot. The internal reference is GAPDH. **I** and **J-** ALP staining (**I**) and ALP activity (**J**). hBMSCs cultured in osteogenic medium (OM) or growth medium (GM) for a week. Scale bar of microscopic images, 100 μm. **K** and **L-** ARS staining (**K**) and ARS quantification (**L**). hBMSCs were grown in an osteogenic medium (OM) or growth medium (GM) for two weeks. Scale bar of microscopical images, 100 μm. **M-O-** Correlation of the expression of KCNMA1-AS1 with that of COL1A1 (**M**), RUNX2 (**N**), and OSX (**O**) during osteogenic differentiation. ns: not significant, *P < 0.05, **P < 0.01, ***P < 0.001, ****P < 0.0001, compared with 0 day/GM.

As previously described in our research(Gu, Jiang et al. 2022), 4 out of 5 lncRNAs were significantly upregulated after osteogenic differentiation according to qPCR results, and KCNMA1-AS1 caught our attention being the leading lncRNA among them (Fig. 1A). Therefore, KCNMA1-AS1 was selected to be used in further study.

To probe into the effects of KCNMA1-AS1 on hBMSCs’ osteogenic differentiation, we studied the expression profile of KCNMA1-AS1 after the osteogenic induction of hBMSCs. Results from qPCR suggested the KCNMA1-AS1 expression levels were significantly elevated during osteogenic differentiation of hBMSCs (Fig. 1B). As expected, the mRNA levels of genes associated with osteogenic differentiation, including collagen type I α1 chain (COL1A1), RUNX2, OSX, and osteopontin (OPN), were significantly elevated during the osteogenic differentiation (Fig. 1C-F). Similarly, the western blot detected elevated protein levels of COL1A1, RUNX2, OSX, and OPN (Fig. 1G and H). Besides, the activity of ALP was found to have increased significantly on the 7th day after the osteogenic induction (Fig. 1I and J). Consistently, after two weeks of culture in osteogenic media, there was also an increase in the number of mineralized nodules that were determined by ARS staining and quantification (Fig. 1K and L). Moreover, the expression patterns of KCNMA1-AS1, COL1A1, RUNX2, and OSX were found to be positively correlated (Fig. 1M-O). The findings from these investigations showed that KCNMA1-AS1 is crucial for hBMSCs to differentiate into osteoblasts.

### KCNMA1-AS1 promotes osteogenic differentiation of hBMSCs in vitro

**Fig. 2:**
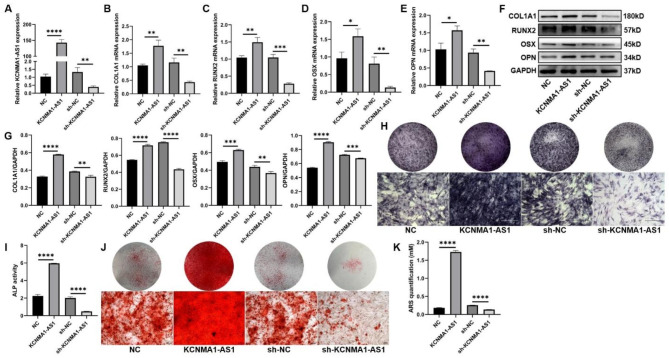
KCNMA1-AS1 promotes osteogenic differentiation of hBMSCs in vitro. **A-** Transfection efficiency of KCNMA1-AS1 overexpression and KCNMA1-AS1 knockdown was measured by qPCR, normalized to GAPDH. **B-E-** Relative mRNA levels of COL1A1 (**B**), RUNX2 (**C**), OSX (**D**), and OPN (**E**) in hBMSCs transfected with lentivirus measured by qPCR after one week of osteogenic induction, normalized to GAPDH. **F** and **G-** The protein levels of COLA1, RUNX2, OSX, and OPN in hBMSCs transfected with lentivirus detected by western blot after one week of osteogenic induction. GAPDH was used as the internal reference. **H** and **I** ALP staining (**H**) and ALP activity (**I**) in hBMSCs transfected with lentivirus after one week of osteogenic induction. Scale bar of microscopical images, 100 μm. **J** and **K-** ARS staining (**J**) and ARS quantification (**K**) in hBMSCs transfected with lentivirus after two weeks of osteogenic induction. Scale bar of microscopical images, 100 μm. *P < 0.05, **P < 0.01, ***P < 0.001, ****P < 0.0001, in comparison to NC/sh-NC.

To investigate the function of KCNMA1-AS1 in the osteogenic differentiation of hBMSCs, overexpression and knockdown models of KCNMA1-AS1 in hBMSCs were developed using lentivirus transfection. Transfection efficiency was evaluated using qPCR assays. The protein expression of KCNMA1-AS1 was remarkably upregulated with KCNMA1-AS1 overexpression, while it was downregulated significantly following KCNMA1-AS1 knockdown (Fig. 2A). To begin with, qPCR results suggested that mRNA levels of osteogenesis-associated genes like COL1A1, RUNX2, OSX, and OPN were elevated after KCNMA1-AS1 overexpression; conversely, the mRNA levels decreased by KCNMA1-AS1 knockdown (Fig. 2B-E). Western blots suggested that KCNMA1-AS1 overexpression improved the expression of COL1A1, RUNX2, OSX, and OPN, while KCNMA1-AS1 knockdown resulted in the opposite (Fig. 2F and G). In addition, ALP activity and calcium deposition were enhanced when KCNMA1-AS1 was overexpressed, and they reduced when KCNMA1-AS1 was blocked (Fig. 2H-K). These findings implied a positive role of KCNMA1-AS1 in regulating in vitro osteogenic differentiation of hBMSCs.

### KCNMA1-AS1 promotes bone formation from hBMSCs in vivo

**Fig. 3:**
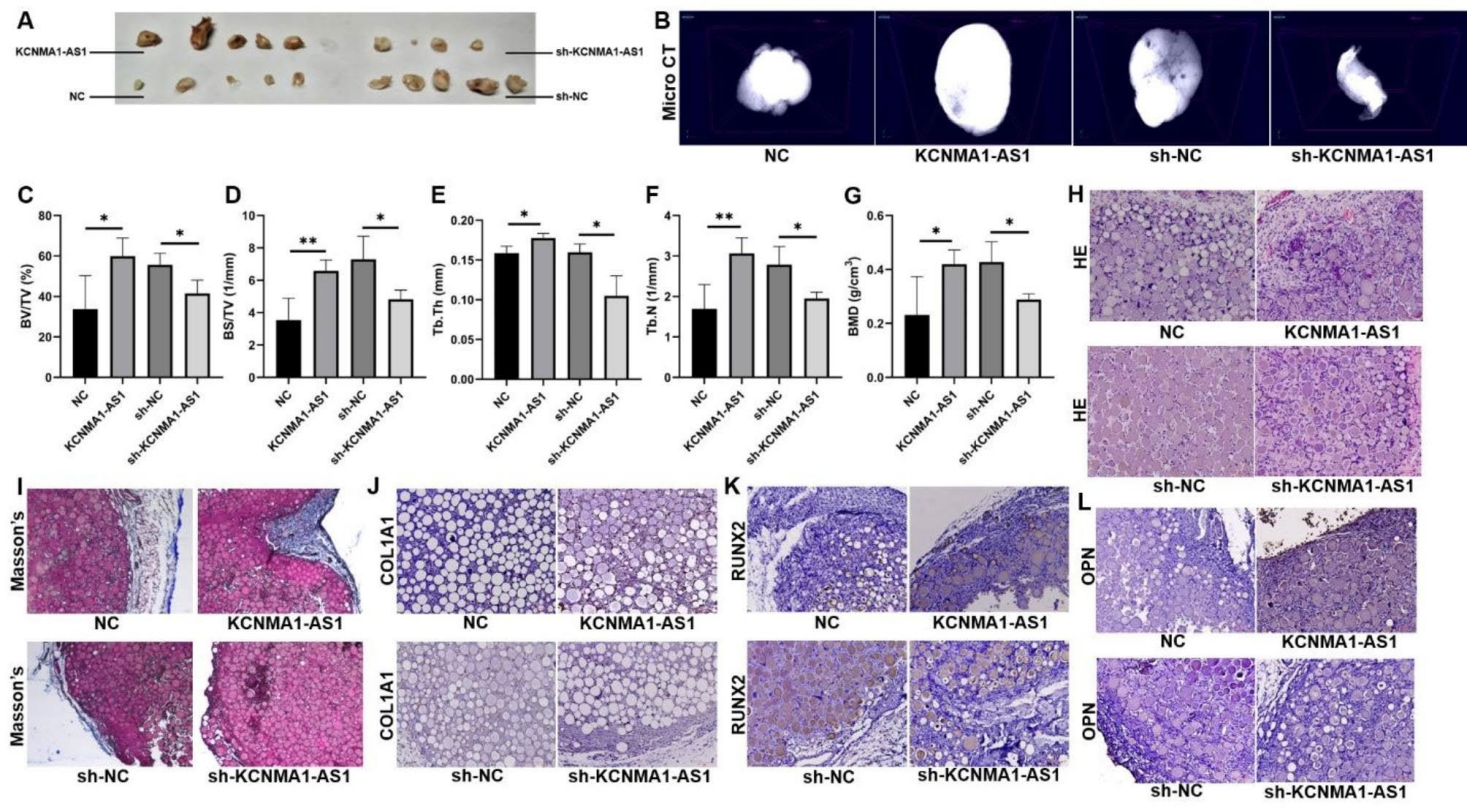
KCNMA1-AS1 promotes in vivo bone formation from hBMSCs. **A-** Xenograft tissue removed from the nude mice. **B-** Representative Micro CT scanning images of xenograft tissues. Scale bar, 500 μm. **C-G-** Bone volume or tissue volume (BV/TV) (**C**), bone surface/ tissue volume (BS/TV) (**D**), trabecular thickness (Tb.Th) (**E**), trabecular number (Tb.N) (**F**) and bone mineral density (BMD) (**G**) analyzed in xenograft tissues. **H** and **I-** HE staining (**H**) and Masson’s trichrome staining (**I**) of xenograft tissues. Scale bar, 50 μm. **J-L**- The expression levels of COL1A1 (**J**), RUNX2 (**K**), and OPN (**L**) in xenograft tissues evaluated by immunohistochemistry. Scale bar, 50 μm. *P < 0.05, **P < 0.01, in comparison to NC/sh-NC.

KCNMA1-AS1’s effect on osteogenic differentiation was investigated in vivo using a heterotopic bone formation model in immunodeficient BALB/c nude mice. Compared to the negative control, larger implants were obtained from the nude mice injected with transfected hBMSCs with KCNMA1-AS1 overexpression. Conversely, smaller xenografts were retrieved from mice treated with transfected cells that had the KCNMA1-AS1 silenced compared to scrambled control (Fig. 3A). Xenografts were scanned and analyzed by a Micro CT scanning instrument (Fig. 3B). Results of the Micro CT indicated increased bone volume, bone surface, bone trabeculae, and bone mineral density (BMD) after KCNMA1-AS1 overexpression, whereas they decreased significantly by KCNMA1-AS1 silencing (Fig. 3C-G). The HE and Masson’s trichrome staining revealed that KCNMA1-AS1 overexpression promoted osteoid formation for having more collagen fibers secreted, which were stained blue in Masson’s trichrome staining, while KCNMA1-AS1 elimination attenuated the process (Fig. 3H and I). Furthermore, the expression levels of osteogenesis-related genes (i.e., COL1A1, RUNX2, and OPN) evaluated by immunohistochemistry were increased following KCNMA1-AS1 overexpression and decreased by KCNMA1-AS1 elimination (Fig. 3J-L). All these results solidified the evidence of KCNMA1-AS1 being a stimulator of osteogenic differentiation of hBMSCs in vivo.

### KCNMA1-AS1 directly binds to SMAD9 in hBMSCs

**Fig. 4:**
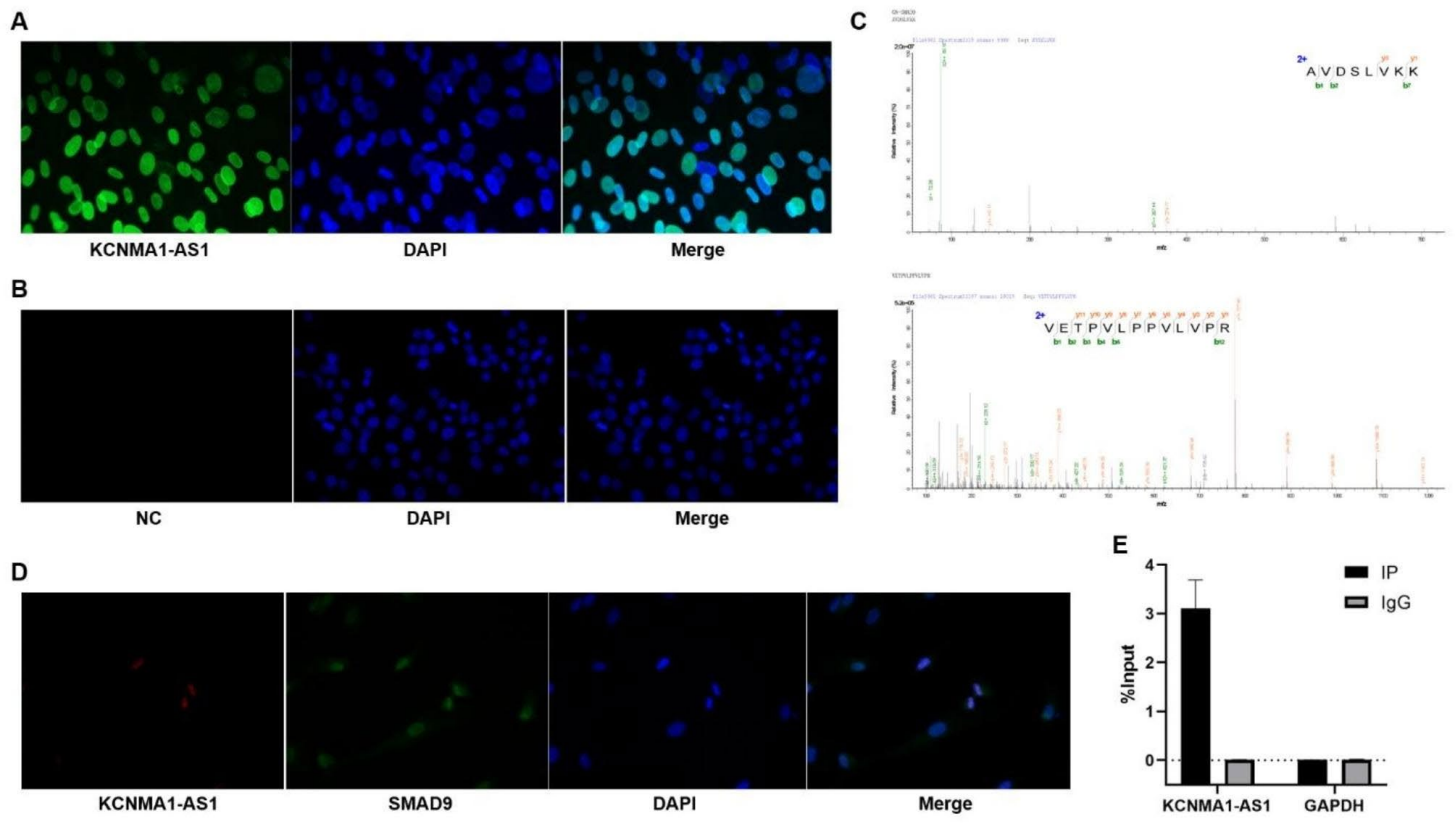
KCNMA1-AS1 directly binds to SMAD9 in hBMSCs. **A and B-** Subcellular localization of KCNMA1-AS1 in hBMSCs detected by the FISH assay. **C-** Mass spectrum of SMAD9. **D-** Colocalization of KCNMA1-AS1 and SMAD9 in hBMSCs detected by FISH assay and immunofluorescence. **E-** RIP assay analyzed by qPCR.

To better understand the underlying mechanism of KCNMA1-AS1 in the osteogenic differentiation of hBMSCs, we first conducted a FISH assay, which revealed that KCNMA1-AS1 was primarily located in the nuclei of hBMSCs (Fig. 4A). It has been demonstrated previously that nuclear-retained lncRNAs can interact with transcription factors or RNA binding proteins (RBPs) to regulate gene expressions and transcriptions(Sun, Hao et al. 2018). Therefore, hBMSCs were transfected with KCNMA1-AS1 overexpression vectors. RNA pull-down assay and mass spectrometry indicated that SMAD9 could be the target of KCNMA1-AS1 (Supplementary Figure S1 and Fig. 4B). Further, FISH and IF assays implied that KCNMA1-AS1 and SMAD9 were co-localized in the nucleus, which provided the spatial basis of their interaction (Fig. 4C). Moreover, RIP assay confirmed that there is a strong connection between KCNMA1-AS1 and SMAD9 (Fig. 4D). Figure 4E shows the IP efficiency of KCNMA1-AS1 by RIP assay. 10% input and IgG were, respectively, as a positive control and a negative control.

### KCNMA1-AS1 activates the SMAD9 signaling pathway during osteogenic differentiation of hBMSCs

**Fig. 5:**
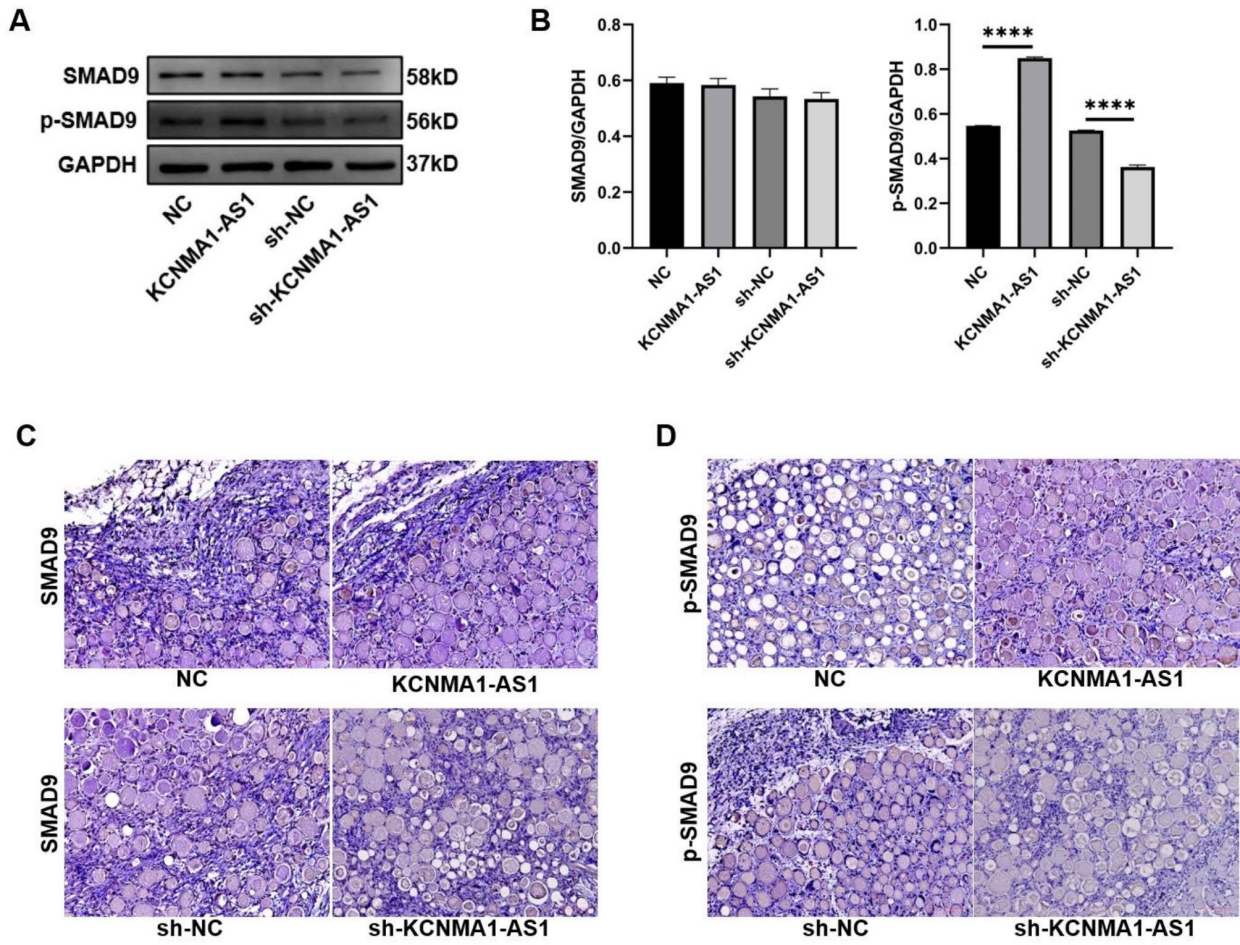
KCNMA1-AS1 activates the SMAD9 signaling pathway when hBMSCs undergo osteogenic differentiation. **A-B-** The SMAD9 protein levels and phosphorylated SMAD9 (p-SMAD9) protein levels in hBMSCs transfected with lentivirus detected by western blot after one week of osteogenic induction. GAPDH served as the reference. **C-D-** The expression of total SMAD9 (**C**) and p-SMAD9 (**D**) in xenograft tissues evaluated by immunohistochemistry. Scale bar, 50 μm. ****P < 0.0001, in comparison to NC/sh-NC.

To understand the relationship between KCNMA1-AS1 and the SMAD9 signaling pathway, hBMSCs were transfected with KCNMA1-AS1 overexpression and KCNMA1-AS1 silenced vectors. Western blots indicated that phosphorylated SMAD9 (p-SMAD9) levels had significantly increased in the KCNMA1-AS1 overexpression model, while it was downregulated following KCNMA1-AS1 knockdown. The protein levels of total SMAD9 remained unchanged in both the KCNMA1-AS1 overexpression and the KCNMA1-AS1 knockdown models (Fig. 5A and B). Similarly, in vivo, IHC results exhibited that the p-SMAD9 expression level was elevated in the xenografts of KCNMA1-AS1 overexpression, while it was decreased in the KCNMA1-AS1 knockdown models. There was no significant difference in the expression of total SMAD9 following both KCNMA1-AS1 overexpression and depletion (Fig. 5C and D). These findings suggest that KCNMA1-AS1 triggers the SMAD9 signaling pathway both in vivo and in vitro, thereby promoting the osteogenic differentiation that was present in the hBMSCs.

### Effect of SMAD9 signaling pathway activated by KCNMA1-AS1 on osteogenic differentiation of hBMSCs

**Fig. 6:**
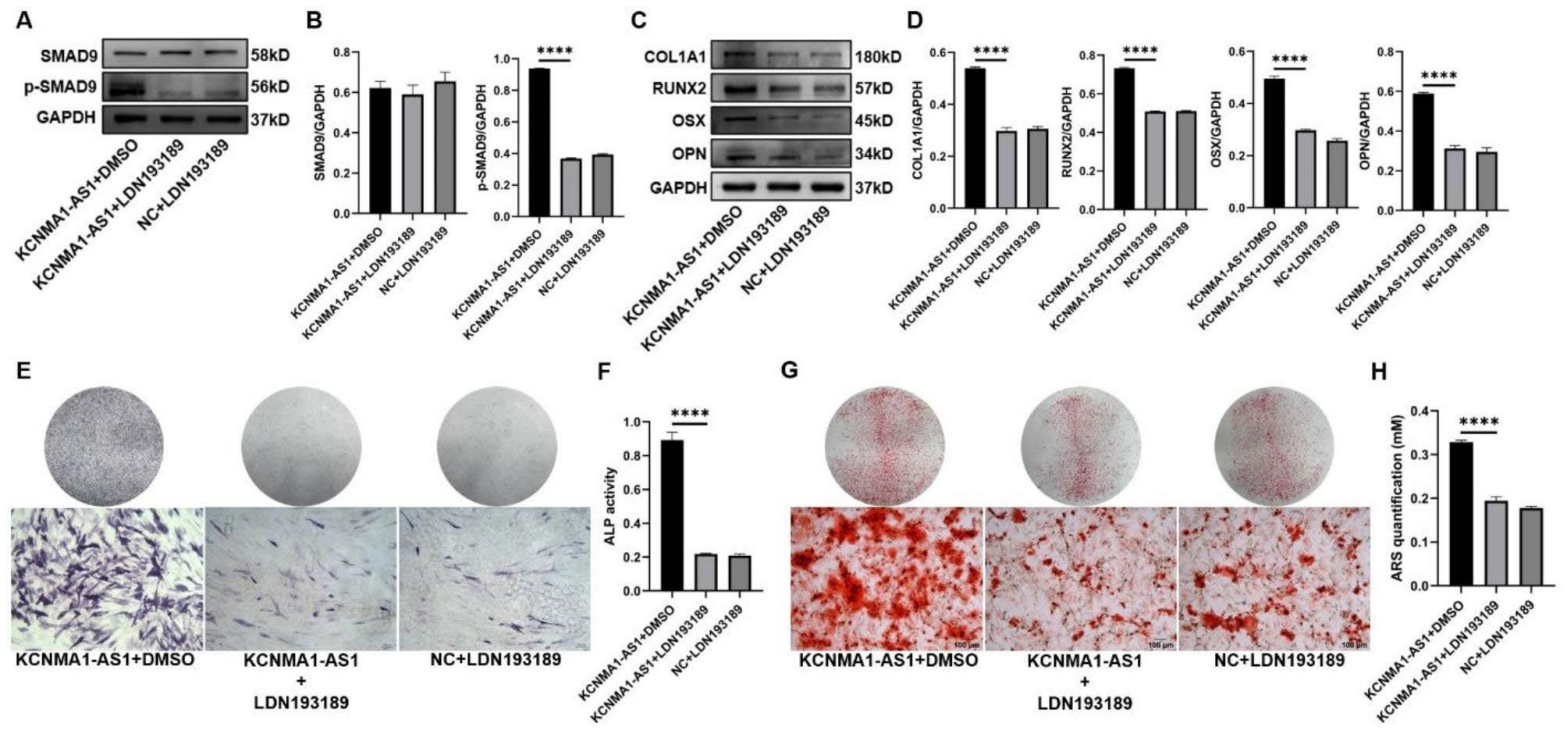
Effect of KCNMA1-AS1 on osteogenic differentiation of hBMSCs with the SMAD9 signaling pathway activation. **A** and **B-** Protein levels of total SMAD9 and p-SMAD9 in lentivirus-transfected hBMSCs treated with DMSO or LDN193189 (100nM) detected by western blot after one week of osteogenic induction. GAPDH as the internal reference. **C** and **D-** The expression levels of COL1A1, RUNX2, OSX, and OPN in lentivirus-transfected hBMSCs treated with DMSO or LDN193189 (100nM) detected by western blot after one week of osteogenic induction. GAPDH as the reference. **E** and **F-** ALP staining (**E**) and ALP activity (**F**) in lentivirus-transfected hBMSCs treated with DMSO or LDN193189 (100nM) after one week of osteogenic induction. Scale bar of microscopical images, 100 μm. **G** and **H-** ARS staining (**G**) and ARS quantification (**H**) in lentivirus-transfected hBMSCs treated with DMSO or LDN193189 (100nM) after 14 days of osteogenic induction. Scale bar of microscopical images, 100 μm. ****P < 0.0001, compared with KCNMA1-AS1 + DMSO.

To further verify whether KCNMA1-AS1 accelerates the osteogenic differentiation of hBMSCs by activating the SMAD9 signaling pathway, we used LDN193189 (GlpBio, USA) as an inhibitor of the SMAD9 signaling pathway. hBMSCs transfected with KCNMA1-AS1 overexpression and negative control was treated with LDN193189. Western blotting of these proteins revealed that the expression of p-SMAD9 was downregulated significantly when hBMSCs underwent LDN193189 treatment, while no apparent alterations were found in the total SMAD9 levels (Fig. 6A and B). Besides, the osteogenesis-related proteins (COL1A1, RUNX2, OSX, and OPN) were found to have substantially deregulated with the influence of LDN193189 (Fig. 6C and D). Administration of LDN193189 counteracted the increased expression of KCNMA1-AS1 on ALP staining and ALP activity (Fig. 6E and F). Equally, ARS staining and ARS quantification indicated that LDN193189 treatment compromised the enhanced effect of KCNMA1-AS1 overexpression on mineralization (Fig. 6G and H). Significant variations were not observed in the expression of total SMAD9, p-SMAD9, osteogenic markers, ALP activity, and mineralization between the KCNMA1-AS1 + LDN193189 group and the NC + LDN193189 group. Overall, these results certainly signify that KCNMA1-AS1 facilitates osteogenic differentiation of hBMSCs by stimulating the SMAD9 signaling pathway.

## Discussion

Successful recovery of bone tissue in large bone defects of the maxillofacial area remains a significant clinical challenge. Driven by urgent clinical demands, developing alternative remedies with the same efficacy as bone autografts and allografts, but without their shortages is important. Exploring the underlying mechanisms involved in the osteogenic differentiation of hBMSCs can provide novel strategies for treatment and meet the pressing demands. Some prior studies have successfully demonstrated that lncRNAs are involved in osteogenic differentiation, indicating their essential involvement in regulating the osteogenesis of hBMSCs [27, 40]. KCNMA1-AS1 was previously shown to possess oncogenic activity and promote the proliferation of ovarian cancer cells [25]. No relevant reports to date have reported KCNMA1-AS1 to have any effect on osteogenic differentiation. In the current study, the increased expression level of KCNMA1-AS1 was observed during the osteogenic differentiation of hBMSCs. Accordingly, the prospective function of KCNMA1-AS1 in regulating osteogenic differentiation was investigated during this work. Gain/loss function assays were performed with lentiviral transfection models in subsequent experiments, which revealed that KCNMA1-AS1 overexpression promoted osteogenesis of hBMSCs both in vivo and in vitro. In contrast,KCNMA1-AS1 deficiency gave rise to the opposite outcome. These results indicated a positive influence of KCNMA1-AS1 on osteogenic differentiation taking place in the hBMSCs.

Further, the underlying osteogenic differentiation mechanism involving KCNMA1-AS1 was investigated using FISH assay and the results indicated that KCNMA1-AS1 was mainly localized in the nuclei of hBMSCs. The function of lncRNAs depends on subcellular localization. Nuclear lncRNAs have been found to influence gene expressions by interacting with transcription factors and proteins in diverse biological processes [41]. For instance, the interaction between lncRNA HOTTIP and WDR5 results in the activation of the Wnt/β-catenin pathway, which enhances the osteogenic differentiation of BMSCs [42]. LncRNA MEG3 regulates chondrogenic differentiation by inhibiting TRIB2, which is achieved by binding with EZH2 [43] as well as LINC02273; it is associated with hnRNPL, which promotes metastasis of breast cancer by increasing AGR2 transcription [44]. Furthermore, using RNA pull-down assay, mass spectrometry, and RIP assay, we were able to detect a tight junction between KCNMA1-AS1 and SMAD9.

Belonging to the transforming growth factor beta (TGF-β) superfamily, mothers against decapentaplegic (SMAD) proteins, which have found eight members in mammals, have been divided into three subgroups, namely, common SMAD (Co-SMAD or SMAD4), receptor-regulated SMADs or R-SMADs, and inhibitory SMADs or I-SMADs. SMAD9 (previously known as SMAD8), being an R-SMAD, is a prominent transcription factor. Also, SMAD9, along with SMAD1 and SMAD5, is phosphorylated and activated directly by BMP type I receptor to form a heterotrimeric complex with SMAD4; these complexes take part in the regulation of target genes and proteins [45–47]. It is important to note that the SMAD9 signaling pathway plays a significant role in regulating bone development as described before [48–51]. Rare lncRNAs have also been reported to be associated with the SMAD9 signaling pathway during osteogenic differentiation, in which lncRNA SNHG5 [52] is an example. Surprisingly, in this study, we discovered that KCNMA1-AS1 overexpression triggered the phosphorylation of SMAD9 both in vivo and in vitro activating the SMAD9 signaling cascade, whereas KCNMA1-AS1 interference attenuated the expression of p-SMAD9, thereby inhibiting the SMAD9 signaling pathway. To verify whether KCNMA1-AS1 impacts the osteogenic differentiation of hBMSCs by activating the SMAD9 signaling pathway, we performed rescue experiments using LDN193189 as a repressor of the SMAD9 signaling pathway. The expression levels of p-SMAD9 and osteogenic specific markers tested by western blot suggested that they were remarkably lowered in hBMSCs treated with KCNMA1-AS1 overexpression and LDN193189 compared to those treated with KCNMA1-AS1 overexpression and DMSO. In addition, the application of LDN193189 abrogated the promotional effects of KCNMA1-AS1 overexpression on ALP activity and mineralized calcium nodules deposition. These findings proved that KCNMA1-AS1 regulates osteogenic differentiation by modulating the SMAD9 signaling pathway. However, the effect of KCNMA1-AS1 on bone regeneration certainly needs to be elucidated further using bone defect models.

The present research showed that lncRNA KCNMA1-AS1 promoted osteogenic differentiation of HBMSCs by targeting the SMAD signaling pathway. LncRNA KCNMA1-AS1 was previously found to be implicated in the progression and migration of epithelial ovarian cancer by promoting proliferation, migration and inhibiting apoptosis [53]. Some other lncRNAs also exhibit a concerning duality - promoting osteoblast differentiation while also fueling cancer progression. Taking an example of LncRNA HOTTIP, it was found to enhance human osteogenic BMSCs differentiation via the activation of the Wnt/β-catenin signalling pathway [42], the activation of which induces enhanced epithelial-mesenchymal transition in cancer metastasis [54]. LncRNA HOTTIP also promoted pancreatic cancer cell proliferation, survival and migration [55]. Taking another example, LncRNA SNHG5 was shown to promote the osteogenic differentiation of BMSCs via the miR-212-3p/GDF5/SMAD pathway. SMAD signaling collaborates with oncogenic pathways like Wnt, NF-kB, and Notch to fuel epithelial-to-mesenchymal transition (EMT) and invasion. LncRNA SNHG5 was also found to play a tumor-promoting role in many cancer types including nasopharyngeal carcinoma [56], hepatocellular carcinoma [57], and cervical cancer [58]. The complex, context-specific functionality of these lncRNAs presents challenges for exploiting their bone regenerative potential while avoiding cancer promotion. There are a few key considerations when assessing whether lncRNAs implicated in both osteogenic differentiation and cancer progression could realistically be used as therapy for bone defects. The complexity of lncRNAs functioning divergently in different cell contexts, their specific roles in driving cancer progression, challenges with targeted delivery, and safety risks of potentially promoting tumors all pose significant barriers to clinical use. Innovative delivery methods, genetic screening, combination therapies with other bone anabolics, short-term treatment and extensive preclinical testing in bone defects models may help mitigate concerns, but far more research is needed. Close collaboration between bone biology and oncology experts will be critical to determine if any lncRNAs implicated in cancer may realistically be harnessed safely and effectively for bone defect therapies. Overall, the cancer risk poses a significant hurdle, and extensive further study is required before this novel genetic targeting approach could be clinically viable.

**Fig. 7:**
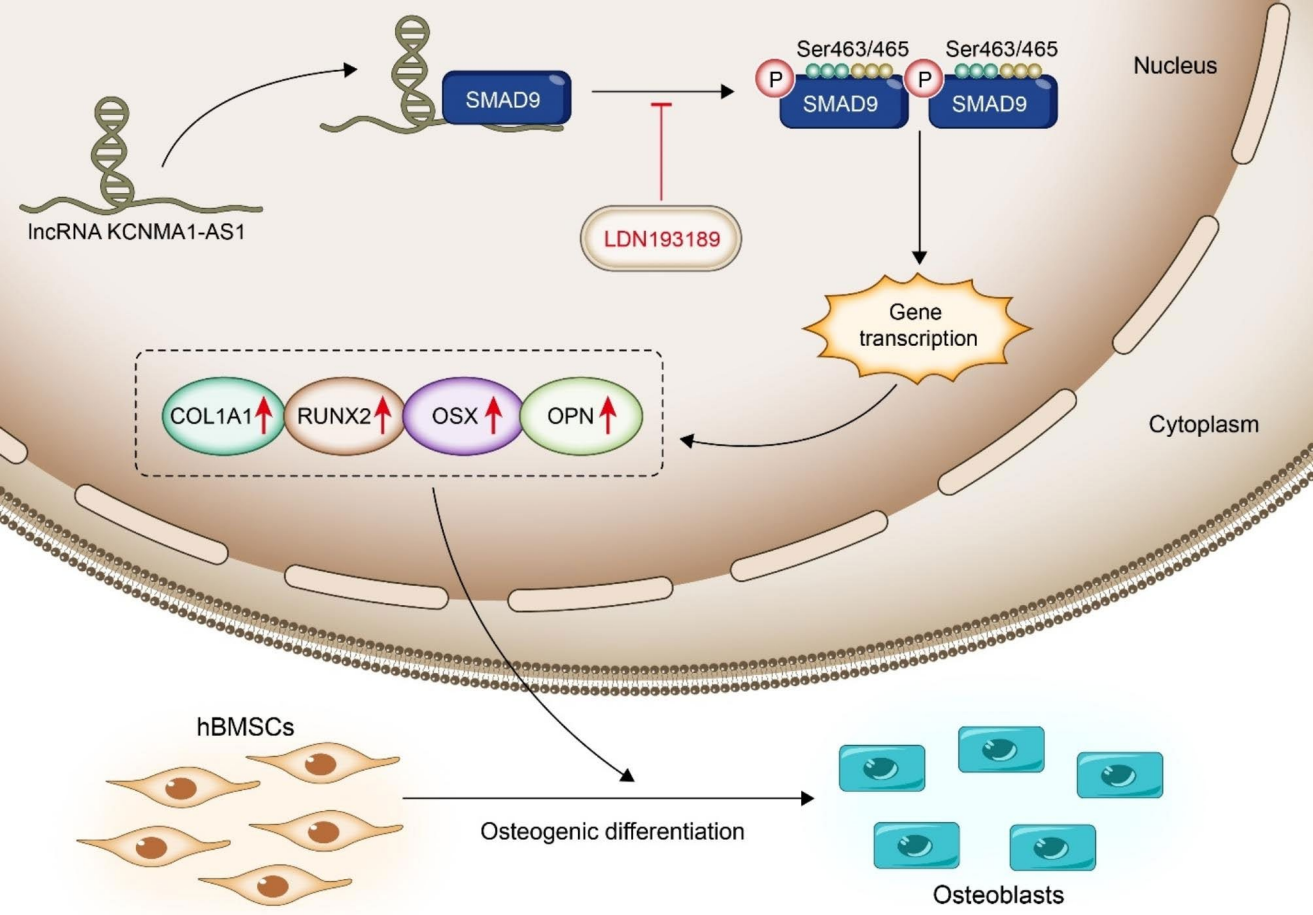
Schematic diagram illustrating the regulation of osteogenic differentiation in hBMSCs by KCNMA1-AS1.

In summary, for the first time, we have reported here that lncRNA KCNMA1-AS1 has augmented expression during osteogenic differentiation. The overexpression of KCNMA1-AS1 results in enhanced hBMSCs’ osteogenic differentiation. Our research revealed a novel mechanism where the KCNMA1-AS1 was found to regulate osteogenic differentiation of hBMSCs via the SMAD9 signaling pathway (Fig. 7), which provides a novel perspective on translational research in bone tissue engineering.

### Electronic supplementary material

Below is the link to the electronic supplementary material.

Supplementary Material 1

## Data Availability

The data used and analyzed during the current study are available from the corresponding author upon reasonable request.
