# Supplementary material for: Long noncoding RNA KCNMA1-AS1 promotes osteogenic differentiation of human bone marrow mesenchymal stem cells by activating the SMAD9 signaling pathway
Source: Biol Direct. 2023 Nov 29;18:81. doi: 10.1186/s13062-023-00425-2 (PMC10685465; doi:10.1186/s13062-023-00425-2)
Supplement: Supplementary file 1 — Supplementary Material 1 [file 13062_2023_425_MOESM1_ESM.docx]

**Table S1** Sequences of primers for qRT-PCR

| Gene | Sequence (5’-3’) |
| --- | --- |
| H-KCNMA1-AS1 | Forward Primer: ATGTTCTTCCCAACCTGCCAA  Reverse Primer: CTCAAACACGAGCGGACCAG |
| H-COL1A1 | Forward Primer: GCCAAGACGAAGACATCCCA  Reverse Primer: GGCAGTTCTTGGTCTCGTCA |
| H-RUNX2 | Forward Primer: TGGTTACTGTCATGGCGGGTA  Reverse Primer: TCTCAGATCGTTGAACCTTGCTA |
| H-OSX | Forward Primer: CCTCTGCGGGACTCAACAAC  Reverse Primer: AGCCCATTAGTGCTTGTAAAGG |
| H-OPN | Forward Primer: AGACCCTTCCAAGTAAGTCC  Reverse Primer: TCATCTACATCATCAGAGTCGT |
| H-GAPDH | Forward Primer: GCACCGTCAAGGCTGAGAAC  Reverse Primer: TGGTGAAGACGCCAGTGGA |

**Table S2** Sequences of siRNA

| Gene | Sequence (5’-3’) |
| --- | --- |
| si-KCNMA1-AS1 | sense: GCUGGAGCUUACAAAGCAAUA  antisense: UUGCUUUGUAAGCUCCAGCUG |
| si-NC | sense: UUCUCCGAACGUGUCACGUTT  antisense: ACGUGACACGUUCGGAGAATT |

**Table S3** Sequences of probes for FISH (FAM and TAMRA labeled)

| Gene | Sequence (5’-3’) |
| --- | --- |
| KCNMA1-AS1 | AGGTGATGCTGACCTGTGTGGCCTAAGGACCACACTTGGTGACCACTGCTCTAAGTGAAACAGCGTGA |

**Table S4** Sequences of probe for RNA pull-down assay

| Gene | Sequence (5’-3’) |
| --- | --- |
| KCNMA1-AS1 | ACCCAGACCTACTGAATTGGAAGTGCTGAGGATGGGGTCCAGCAATCTGTGGTTGAACAAGCTCTGCAGGCATCCAGGAGCAGAGCTGTGGGGCAGCAGAATCTGTGATGACAGAGAATGATGATGTATTTTTGCCAGCAGATGCTTTTGCTCCTGTTTTGATACCCCAATATTCGGATGCAGAAAAGGACTCTGAGCATTGCTGACGGGTGTGCTCTGCACACCTATCTCTTCAGGATCTGAATAATTTGGTGGAAAGACAATGCCTTTTCTGGAAAAGGGTGTCAGGAAAGGTCCAAGGCTCAAGGAAAATGGACATCATGTGCACAACCTCCTAACTCAGAGGACAAGCAAGTGACCGGAAGGCCAAGGATGCTGTCTGACCACAGACATTGCAATGCAGACACATAAGATTAGAAGCAAAACGCAGGAGGCTGGAGTTACCTTTCATGGAAGGGTAAGCAGGAAGCAACTACAAGATCATCATTTCAAAACAATATGAAAGCAGAATGGGAAATGCCATGGCATGCTCCCTCTTTTCAGGCAATATGGACACTTTGGGGAAGTTATGAAGCGTCTCCCATTCCCGCTTGAGGTACTCAATAGAGCCCACAAACACAATGTGCTTGAGCTCATGGTAATGAAAGTTGCTGGCACGGAGCGGCATCACCAGGTTCCGGAGGCCGATCAGGGCTGAGCTGACGTCGCCAAAGATGCAGACCACGACATGGCCACTCAGGACGGTCATGGCAGCTTCACTTCGAGTCTACAACAGGGAGAAGTGGGTAAGAGTCAGAGAGAAGACTGCATAGGGCTGTTGTAAGGAGTGAAATAAATCATTTGTGCAAAGAATCAAGCAAATA |

Marker Input RNA pull-down NC


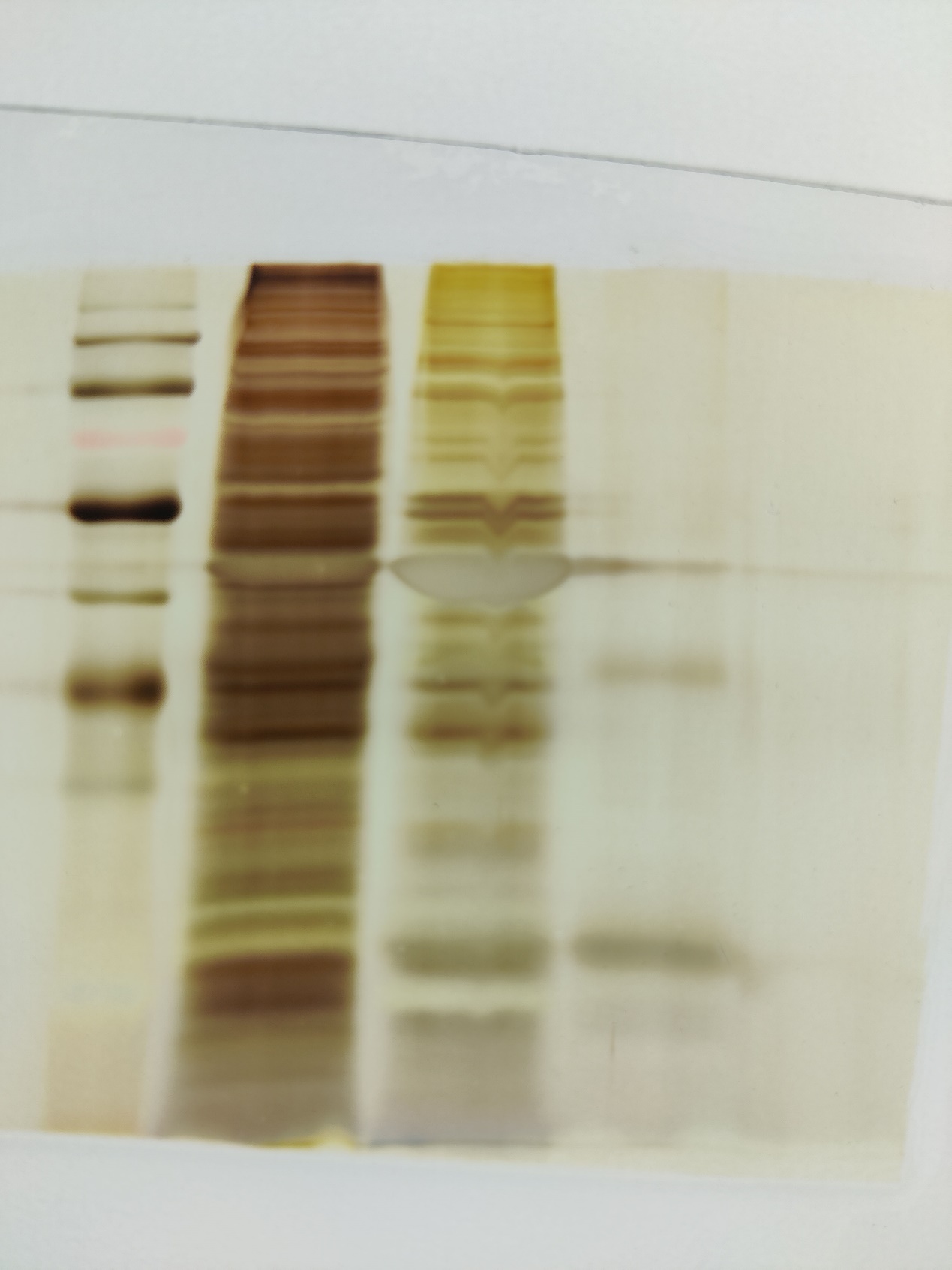


**Figure S1.** Image of silver staining for RNA pull-down assay.

1 2 3 4 5 6 7


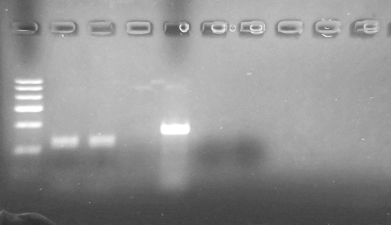


**Figure S2.** Electrophoresis gel of RIP assay. 1: Marker (600/500/400/300/200/100 bp), 2: Input (KCNMA1-AS1), 3: IP (KCNMA1-AS1), 4: NC (KCNMA1-AS1), 5: Input (GAPDH), 6: IP (GAPDH), 7: NC (GAPDH).
